# Statistical iterative reconstruction algorithm for X-ray phase-contrast CT

**DOI:** 10.1038/srep10452

**Published:** 2015-06-12

**Authors:** Dieter Hahn, Pierre Thibault, Andreas Fehringer, Martin Bech, Thomas Koehler, Franz Pfeiffer, Peter B. Noël

**Affiliations:** 1 Chair for Biomedical Physics and Institute for Medical Engineering, Technische Universität München, Munich, Germany; 2 Department of Physics and Astronomy, University College London, United Kingdom; 3 Medical Radiation Physics, Lund University, Lund, Sweden; 4 Philips Technologie GmbH, Innovative Technologies, Hamburg, Germany; 5 Institute for Advanced Study, Technische Universität München, Munich, Germany; 6 Department of Radiology, Technische Universität München, Munich, Germany

## Abstract

Grating-based phase-contrast computed tomography (PCCT) is a promising imaging tool on the horizon for pre-clinical and clinical applications. Until now PCCT has been plagued by strong artifacts when dense materials like bones are present. In this paper, we present a new statistical iterative reconstruction algorithm which overcomes this limitation. It makes use of the fact that an X-ray interferometer provides a conventional absorption as well as a dark-field signal in addition to the phase-contrast signal. The method is based on a statistical iterative reconstruction algorithm utilizing maximum-a-posteriori principles and integrating the statistical properties of the raw data as well as information of dense objects gained from the absorption signal. Reconstruction of a pre-clinical mouse scan illustrates that artifacts caused by bones are significantly reduced and image quality is improved when employing our approach. Especially small structures, which are usually lost because of streaks, are recovered in our results. In comparison with the current state-of-the-art algorithms our approach provides significantly improved image quality with respect to quantitative and qualitative results. In summary, we expect that our new statistical iterative reconstruction method to increase the general usability of PCCT imaging for medical diagnosis apart from applications focused solely on soft tissue visualization.

The limited ability to record the full dynamic range of a signal is a constant concern in imaging. In photography for instance, the camera sensor is most often unable to capture the full contrast of a scene, forcing the photographer to find a compromise between underexposed and saturated regions. Extension of the dynamic range can be achieved, among other ways, through a non-linear transformation of the signal or by combining multiple photographs taken with different exposure times. The non-linear approach typically involves important degradation of the signal-to-noise ratio, while the multiple-capture approach can lead to difficulties caused by subject motion or imperfect registration of the individual snapshots.

Difficulties related to limited dynamic range occur, in one form or the other, in all other branches of imaging. Conventional X-ray radiography relies on the attenuation of X-rays to produce contrast. Thanks to the negative exponential response of the attenuation with the integrated thickness of an object or a subject (known as the Beer-Lambert law), the signal of interest is naturally “compressed” non-linearly in transmission values that range from 100% to 0%. In this case, problems arise when highly absorbing elements - typically metallic implants in medical applications -- cause such high absorption that the transmitted X-rays are reduced to an undetectable level. When radiographs are combined for three-dimensional imaging, such implants usually lead to streak artifacts in computed tomographic reconstructions, an effect commonly termed “metal artifacts” in the literature^1^,^2^,^3^,^4^,^5^,^6^. Similarly, sufficient contrast in soft tissue is not easily achieved with conventional computed tomography (CT) because of their weak absorption relative to bone of small variations of the attenuation coefficients between the elements that constitute soft tissue.

Higher soft-tissue contrast can be obtained with phase sensitive imaging methods^7^. These methods make use of the phase shift incurred to X-rays, instead of their absorption, as they pass through matter. Phase-contrast imaging leads to a soft-tissue contrast that is significantly higher than in absorption imaging^8^,^9^,^10^,^11^,^12^ and is able to deliver additional and complementary information^13^,^14^, owing to the fact that the variations in refraction between different soft-tissue materials are by orders of magnitude larger than those of the attenuation coefficients^15^,^16^. However, this high sensitivity to small density variations leads to a problem similar to the effects that metal has in conventional CT, this time manifesting itself as phase wrapping. Phase wrapping occurs in all phase-contrast techniques, as the quantity that is measured—the phase shift—is defined on the unit circle, i.e. in the interval [− *π*,*π*]. If the phase shift of the X-rays is outside this interval, the value is wrapped back into this interval and thus leads to no longer uniquely defined measurements. Such a phenomenon usually happens when the X-rays are passing through dense objects, such as bones, and produces artifacts similar to those caused by metal in conventional CT, termed ‘bone artifacts’.

One of the most successful X-ray phase-contrast imaging techniques developed over the last decade is based on grating interferometry^17^,^18^,^19^,^20^. Grating interferometry imaging can work with standard laboratory sources^21^,^22^, possibly in compact setups^23^, making it especially promising for clinical application. In addition to the phase-contrast signal, the technique provides a conventional absorption image, as well as a dark-field signal, which maps small angle scattering inside an object^24^,^25^,^26^,^27^ (see fig. 1). These three different signals are measured and extracted simultaneously and are therefore naturally perfectly registered.

Initial investigations have illustrated the high potential of iterative as well as advanced reconstruction but also the need for specific algorithms which are designed for PCCT^28^,^29^. In this letter we show how it is possible to extend the effective dynamic range of a reconstructed tomographic volume through a combination of the three signals provided by a grating interferometry imaging system. Our approach eliminates most of the artifacts originating from dense objects. The soft-tissue regions, to which the phase-contrast signal is sensitive, are combined with information on the location of the dense parts, e.g. bones, taken from the absorption signal. The combination is described through a statistical model, modifying the statistical uncertainties which are primarily obtained from the dark-field signal and it is implemented within our in-house developed statistical iterative reconstruction framework.

We demonstrate the capabilities of this new statistical iterative reconstruction (SIR) algorithm with a study of preclinical relevance. The example is the abdominal region of a mouse with bones measured with synchrotron radiation. The results gained with the new algorithm are compared to those obtained with a conventional analytical reconstruction (filtered backprojection (FBP)) and a standard iterative reconstruction (IR) and show significant improvements in image quality and a strong reduction of the number and intensity of bone artifacts.

## The Problem

We identified the following three primary causes that lead to the appearance of bone artifacts:

1. The strong absorption in dense materials leads to photon starvation and loss of information.
2. Small-angle scattering inside dense, porous materials causes a loss of coherence and thus limits the ability to reliably determine the phase shift.
3. The measurement of a phase shift is intrinsically restricted to the interval [−*π*,*π*]. If at any position the gradient exceeds this range it will be wrapped back into this interval. This phase wrapping usually occurs at strong edges, where refraction and thus the differential phase shift is especially high, e.g. at the boundary between soft tissue and bones. In combination with 1., measurements close to the boundaries of the phase gradient interval can become wrapped due to higher noise fluctuations at positions where the count rate is lowered by strong absorption. This phenomenon is called statistical phase wrapping^30^,^31^.

All of the above effects lead to a phase signal that is no longer uniquely defined at certain positions and thus does not represent reliable information for the tomographic reconstruction.

## Results

Unlike the case of reduction of metal artifacts in conventional CT, PCCT can make use of the three signals (phase-contrast, absorption, dark-field), which are naturally perfectly registered, to overcome the limited dynamic range problem occurring with dense objects.

### Description of the algorithm

The first part of the reconstruction scheme is the signal extraction from the raw interferometer projections. This task is done in a weighted least-squares approach that fits a periodic function onto the stepping curves. The weights are taken directly from the measured intensities in form of the inverse variance of the intensity.

This information is subsequently utilized in the SIR algorithm, which is formulated as the minimization of an objective function (see equation 1 in Methods). The first part of this function is the so-called data-fidelity term, that quantifies how well the reconstructed image fits to the measured data. It takes the form of a weighted least-squares term, containing the difference between the measured DPC projections and a forward model of the reconstruction problem—a projection operator and a derivative to account for the differential input data. This difference is weighted with the variances calculated during the signal extraction step, to control each individual projection pixel’s influence on the final reconstruction. Pixels with a high variance are considered to contain less reliable information and their influence on the data fidelity is reduced accordingly.

**Figure 2:**
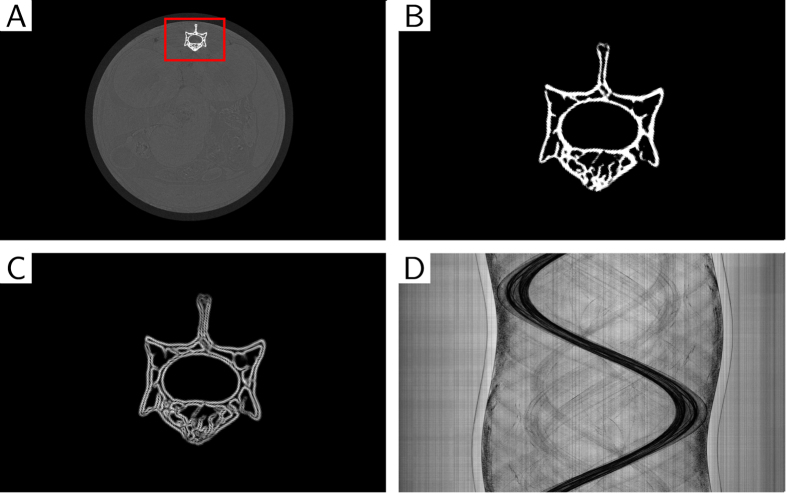
Illustration of the steps necessary for creating the mask *m* for modification of the statistical weights in the cost function. This modification will reduce the effects of phase-wrapping at the transit regions between soft-tissue and dense objects. The mask is initially created from an absorption reconstruction (**A**), where dense regions—the bones—are segmented using a threshold method. In panel (**B**) an enlarged view of the segmented region marked with the red box in (**A**). The threshold was chosen in a way to maintain the full details of the bone. Besides being used in the creation of the weight modification, this segmented image is also directly used as a mask for the bone regularization and its inverse as a mask for the Huber regularization. To gain knowledge on the bone to soft-tissue boundaries, the gradient magnitude of the segmented image is calculated, as shown in panel (**C**). Finally, the gradient image is forward projected, normalized to the interval [0,1] and inverted to create the weight modification *m*. The result of multiplying the modification onto the statistical weights is shown in panel (**D**).

At this point the formulation of the algorithm is very general. Additional steps have to be taken to reduce phase wrapping artifacts caused by dense objects. We address the limited dynamic range problem by restricting the influence of presumably phase-wrapped pixels on the reconstruction. The locations of pixels with a high probability of being wrapped have to be determined. This is accomplished by making use of the absorption signal, precisely delineating bones or other dense objects. The following steps are performed and illustrated in Fig. 2:

1. reconstruction of absorption signal (Fig. 2(A))
2. dense object / bone segmentation via thresholding method (Fig. 2(B))
3. calculation of 3D gradient magnitude of segmented volume (Fig. 2(C))
4. forward projection of gradient volume
5. combining gradient projections with statistical weights (Fig. 2(D))

By thresholding the absorption reconstruction (step 2), the bones including their inner structures are preserved. The gradient calculation (step 3) is performed to determine the location of tissue-bone boundaries. At these boundaries the phase gradient will be especially large. A forward projection operation of these boundaries is then used to pinpoint the location of likely phase-wrapped pixels in projection space.

Reconstructing with the data fidelity alone does not provide adequate results, because CT or PCCT reconstruction is an ill-posed problem resulting in an unstable solution in the presence of noise. A stable solution is obtained by regularizing or constraining the objective function. Most regularization terms are based on some a-prior knowledge like an expected smoothness of connected regions. A simple quadratic regularization, for example, is defined as the sum of quadratic differences between one voxel and its nearest neighbors. The result is the penalty that is added to the objective function. In the optimization procedure this will lead to reduced value differences between neighboring voxels and thus helps to keep the resulting volume smooth. It should be noted that the expected reduced differences constitute a known prior of the object to be reconstructed. The same principle is applied in edge-preserving regularization, where the quadratic function is replaced with a Huber potential function on the voxel differences. This piecewise-defined function is linear for differences above a choosable threshold and quadratic below to further smooth out already flat regions. In this work, the Huber term is applied with a mask to restrict its effect only to certain parts of the volume. For the treatment of the bone regions, a novel regularization term was designed. It is defined as the quadratic difference between a voxel of the phase reconstruction and the corresponding voxel of the absorption reconstruction. If a constant factor for bone material is assumed, this term forces the phase values corresponding to bone material towards realistic values, whereas before they were unreliable due to dynamic range problem in the projection data, and effectively couples both signals. A similar regularizer has been used in propagation-based phase contrast imaging^32^. Just as the Huber term, the bone regularization contains a mask to restrict its effect to parts of the volume.

### Experimental verification

An ex-vivo phase-contrast CT of a formalin-fixed mouse was measured with a grating interferometer installed at beamline ID19 of the European Synchrotron Radiation Facility (ESRF). More information can be found in the Materials and Methods section and in^33^. The dataset was subsequently reconstructed using a standard PCCT FBP^21^, IR and our SIR algorithm.

The statistical weights resulting from the least-squares processing step were modified using the procedure as explained in the previous section and are illustrated in fig. 2, using the absorption signal retrieved from the same measurement. The quadratic regularization term was applied to the complete volume, whereas the Huber and bone regularization were only employed at complementary regions: a mask (Fig. 2B) was used to determine the non-overlapping regions (bone vs. soft-tissue) where the Huber or bone regularization are employed. More details on the reconstruction are given in the Methods.

**Figure 3:**
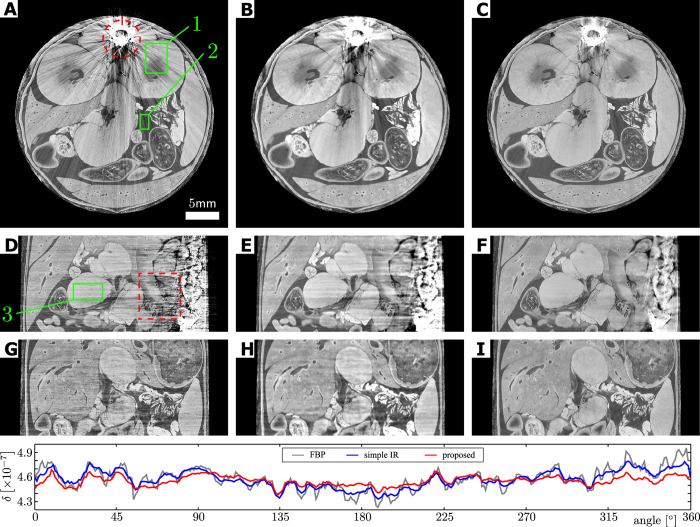
Results of reconstructions from an ex-vivo mouse X-ray phase-contrast CT measurement using FBP (left column), standard IR (middle column) and the proposed sSIR algorithm (right column) in the form of axial slices (**A**, **B**, **C**), as well as sagittal (**D**, **E**, **F**) and coronal (**G**, **H**, **I**) cuts through the center of the volume. When comparing the FBP results with results from our proposed algorithm, one can observe that the strong streaking artifacts and the shadowing around the bone in the FBP (**A**) as well as in the IR reconstruction (**B**) are clearly reduced in (**C**). In the sagittal view (**D**) the streaking artifacts lead to vertical lines which are strongest in the vicinity of bones. Even relatively far away from the bone the artifacts affect the image quality, as visible in the coronal view (**G**). Both of these effects obstruct the underlying small details, which become much clearer and easier to detect in our SIR reconstructions . A quantitative analysis of the streak reduction is shown on the bottom in the form of a line plot along the red, dashed circle in panel (**A**). This plot demonstrates the amount of artifact reduction possible with our method compared to FBP (gray line) and IR (blue line), while retaining the underlying tissue structure. In addition, several regions-of-interest are placed (green rectangles). The ROI results are given in Table 1.

**Figure 4:**
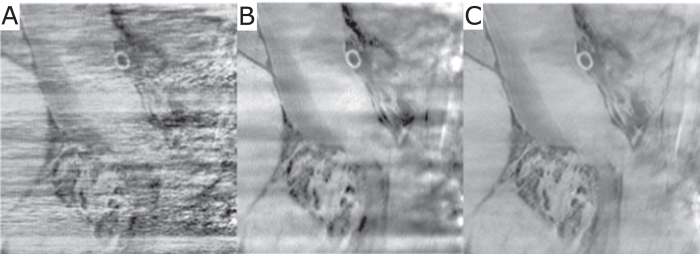
Enlarged view of the sagittal cut from fig. 3(D–F), which is marked with the red dashed rectangle. The region is in the vicinity of bones and demonstrates in more detail the drastic reduction of artifacts overlaying soft-tissue structures when using the proposed algorithm (**C**) compared to FBP (**A**) and IR (**B**).

Figure 3 presents the results of reconstructions performed using FBP (left column), standard IR (middle column) and the proposed SIR algorithm (right column) in the form of axial slices (A, B, C), as well as sagittal (D, E, F) and coronal (G, H, I) cuts through the center of the volume. All six images are windowed in the same range of *δ* = [4.067 × 10^−7^,5.067 × 10^−7^]. As already observed by other investigators, the FBP as well as the standard IR reconstruction suffer from strong streaking artifacts and shadowing around the bone in axial view and a noise-like texture in coronal view, obstructing most of the fine anatomical details. Results from our algorithm show that intensity and extension of bone artifacts are drastically reduced, and that the image quality is significantly improved. This becomes most apparent in the sagittal and coronal views, where the images appear significantly clearer and almost free of artifacts. Figure 4 shows an enlarged view of the part of the sagittal cut marked with a red dashed rectangle in fig. 3(D), and clearly illustrates the amount of artifact reduction and detail visibility with SIR (C) compared to the standard FBP (A) and IR (B).

In order to quantify the reduction of artifacts, the bottom of fig. 3 shows a line plot along the red dashed circle in panel A. The 0° point is marked with a vertical bar on the circle and the data are collected in clockwise order. The gray line corresponds to the FBP, the blue line to the IR and the red line to the proposed SIR algorithm results. This plot shows the capability of the proposed algorithm to reduce streaks, while maintaining the underlying details. Examples for these details can be seen at around 135^°^ and 220^°^, where both curves lie on top of each other, whereas in the other parts of the plot the FBP curve contains many more and stronger variations. In addition, the standard deviation of several regions-of-interest (ROI) were measured. The ROIs are marked with green rectangles in the left column images of fig. 3. The results are summarized in Table 1 and illustrate that a significant reduction of noise by factors of 1.5–2 is achieved.

## Discussion

In this study we presented a new statistical iterative reconstruction algorithm specially designed for PCCT. When employing our algorithm on experimen data a reduction of bone artifacts and a significantly improved image quality can be reported. By the incorporation of all three complementary signals our reconstruction approach enables one to overcome the limited dynamic range problem. This implies that PCCT significantly benefits from our statistical iterative reconstruction algorithm since a wider range of pre-clinical samples can be successfully imaged.

Future work will focus on demonstrations that our algorithm performs well in other sub-optimal imaging scenarios where analytical reconstruction algorithms tend to provide poor image quality. With the experimental data used in this study, our algorithm provided robust and sound results, even considering the fact that both the X-ray energy and the chosen Talbot distance contributed to stronger streak artifacts. Low energy leads to an increased attenuation, which yields information loss. The high Talbot order—that is, a large distance between the phase and absorption grating—produces large transverse shifts of the interference pattern and thus increased the probability of phase wrapping. Proving the possibility of bone artifact reduction in such a difficult measurement setting demonstrates the potentials of our approach in the context of translation to laboratory standard X-ray tubes. The translation to standard X-ray tubes marks an important step toward a successful clinical translation. Furthermore, current clinical standards, for example with respect to radiation exposure and acquisition speed, need to be maintained for a future clinical system. At the same time, the image quality of clinical PCCT FBP reconstructions will be significantly below an acceptable diagnostic demand, especially when bone is present. Thus it is absolutely essential to use an algorithmic solution like the one presented in this work as foundation to accelerate the clinical translation of PCCT. When employed in the clinical arena our applicability to handle and reduce bone artefacts will be of highest importance for any clinical indication.

In summary, we have shown on experimental data that the presented statistical iterative reconstruction algorithm increases the general usability of PCCT imaging for pre-clinical studies apart from applications focused solely on soft tissue visualization. This is a central milestone in transforming grating-based phase-contrast X-ray tomography from an experimental status to a robust and highly usable tool in small-animal imaging. The results of our investigation suggests a possible future clinical translation of PCCT.

## Methods

### Phase-Contrast and Absorption Computed Tomography

The principle of grating-based PCCT and its projection acquisition is explained in detail in^8^,^18^,^19^. The X-rays pass through the object and are attenuated and refracted. The refraction causes a change in the direction of the X-ray path, which can be measured indirectly using an interferometer. The phase-contrast and the absorption-contrast images are acquired simultaneously with this method. The experimental arrangement consists of a phase grating *G*1 and an analyser grating *G*2. The image contrast itself is formed via the combined effect of the two gratings. The second grating (*G*1) acts as a phase mask and imprints periodic phase modulations onto the incoming wave field. Through the Talbot effect, the phase modulation is transformed into an intensity modulation in the plane of *G*2. When one of the gratings is scanned along the transverse direction *x*_*g*_, the signal *I*(*m*,*n*) in each pixel (*m*,*n*) in the detector plane oscillates as function of *x*_*g*_^24^:


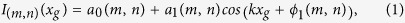


where *a*_*i*_ are the amplitude coefficients, *ϕ*_*i*_ the corresponding phase coefficients, and *k* the period of *G*2. Absorption, phase-contrast, and dark-field images can be obtained as the zero- and first-order components of the Fourier transform, equivalent to fitting the obtained intensity curves *I*_(*m*,*n*)_(*x*_*g*_) to a cosine function or performing a least-square fit of cosine functions. A set of reference images (with superscript *r*) of the empty beam is acquired for normalization of the sample images so that the final images of absorption, phase, and dark-field are calculated by:


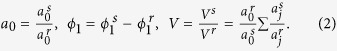


### Experiment details

The abdominal region of a mouse cadaver—fixed in formalin and placed in a plastic container—was measured in a two-grating interferometer installed at beamline ID19 of the European Synchrotron Radiation Facility (ESRF) in Grenoble, France. The interferometer consisted of a phase grating with period and an absorption grating with period and an inter-grating distance of . This distance corresponds to the 9^th^ fractional Talbot order. The measurement was performed with monochromatic X-rays with an energy of 23 keV. The dataset was recorded in 902 projection views with four stepping images each. All images were recorded with a FReLoN camera, a scintillator lens-coupled CCD, with an effective pixel size of and image dimensions of 1453 × 433 pixels. Since this detector type suffers from spatial crosstalk, the raw projection images were deconvolved before the signal extraction step to improve spatial resolution. As the point spread function of the detector system was not exactly known, it was estimated as a two-dimensional Gaussian function with *σ*_*x,y*_ = 1 pixel. To prevent phase wrapping at the edges of the container during the measurement, it was placed in a water bath that extended over the field of view.

### Reconstruction parameters

The reconstruction shown in the left column of fig. 3 was done with a standard filtered backprojection algorithm using a Hilbert filter to handle differential phase-contrast data as described in^22^. The reconstruction in the right column of fig. 3 was performed with our presented statistical iterative algorithm. It consists of optimizing the objective function


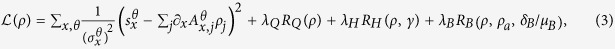


where *s* is the measured projection data, ∂_*x*_*A* is the differential forward projection operator, *ρ* corresponds to the reconstructed quantity, *σ*^2^ represents the variance of the measured data and *λR* are the regularization terms with *Q*, *H* and *B* standing for quadratic, Huber, and bone, respectively. In the last term, the electron density *ρ* is coupled to the absorption density *ρ*_*a*_ through a proportionality constant *δ*_B_ / *μ*_B_ based on the a model for the X-ray index of refraction of bone material. Such a proportionality constant has been used in similar previous experiment^34^.

The optimization was done using a standard nonlinear conjugate gradient algorithm. The regularization strength parameters, as well as the threshold of the Huber term were chosen empirically, such that the result was visually and quantitatively most accurate. The results of both reconstructions were converted to units of the refractive index decrement *δ* by


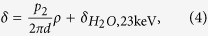


where the addition of the refractive index decrement of water takes into account the offset created by the water bath.

## Additional Information

**How to cite this article**: Hahn, D. *et al.* Statistical iterative reconstruction algorithm for X-ray phase-contrast CT. *Sci. Rep.* **5**, 10452; doi: 10.1038/srep10452 (2015).

## Figures and Tables

**Figure 1:**
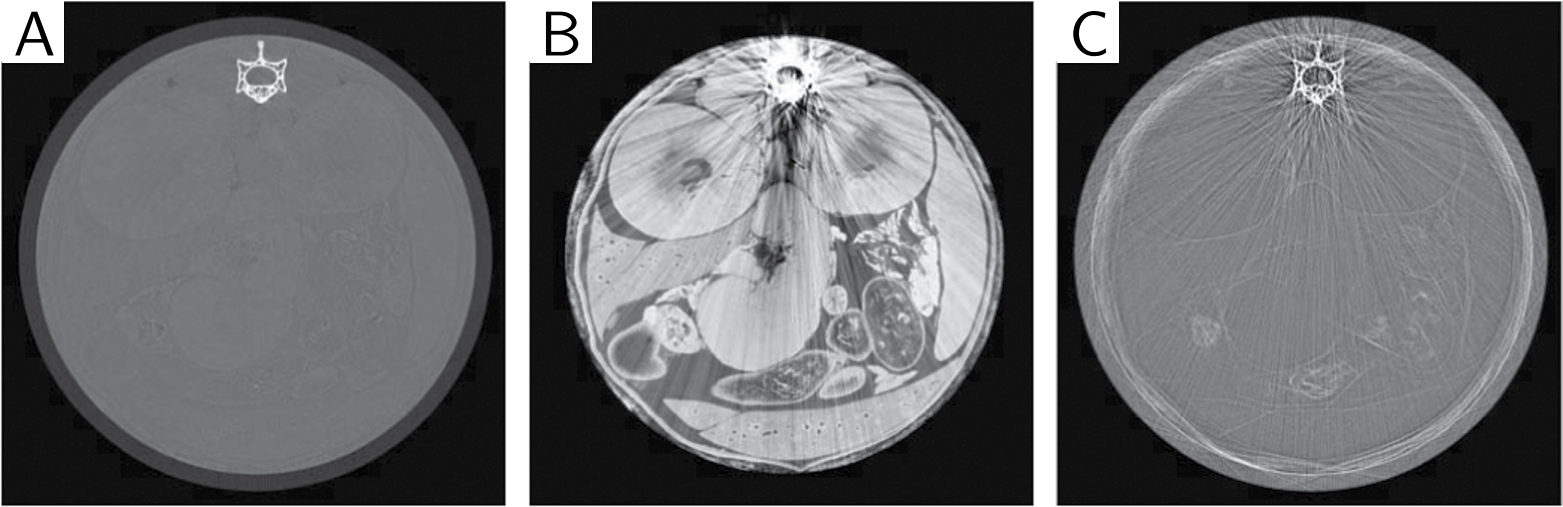
Standard FBP tomographic reconstructions from the three signals ((**A**) absorption, (**B**) phase-contrast, (**C**) dark-field) available from a grating interferometry acquisition. These three different signals are measured and extracted simultaneously and are therefore naturally perfectly registered. The absorption signal allows for an accurate delineation of the bone, but soft-tissue contrast is limited. The phase-contrast reconstruction exhibits strong soft-tissue contrast but problems arise in the vicinity of high density objects. Different information is uncovered with the dark-field reconstruction (small-angle scattering).

**Table 1:** Results of the standard deviation analysis of several regions of interest in fig. 3

region of interest	FBP	IR	SIR	*σ*FBP/*σ*SIR	*σ*IR/*σ*SIR
axial (ROI 1)	5.01 ⋅ 10^−9^	4.22 ⋅ 10^−9^	3.78 ⋅ 10^−9^	1.33	1.12
axial (ROI 2)	2.27 ⋅ 10^−9^	1.85 ⋅ 10^−9^	1.56 ⋅ 10^−9^	1.46	1.39
sagittal (ROI 3)	2.42 ⋅ 10^−9^	2.07 ⋅ 10^−9^	1.16 ⋅ 10^−9^	2.09	1.78
